# Does SGLT2 inhibition modify the inflammatory profile in non-diabetic CKD patients? A new avenue beyond diabetes

**DOI:** 10.1097/MS9.0000000000004672

**Published:** 2026-01-20

**Authors:** Muddassir Khalid, Muhammad Abu Bakar, Muhammad Rizwan Javed, Rabia Narmeen Numani

**Affiliations:** a Department of Medicine, Nishtar Medical University, Multan, Pakistan; b Department of Medicine, Sahiwal Medial College, Sahiwal, Pakistan

The hallmark of progression in chronic kidney disease (CKD) is chronic inflammation, which is related to various factors, including decreased GFR, leading to the accumulation of pro-inflammatory events, such as oxidative stress, endothelial dysfunction, the release of cytokines (IL6 and TNF-alpha), and the activation of fibrosis-related pathways (TGF-beta and NF-kb). Since CKD is a multifactorial disease, it requires a multifaceted approach utilizing both nonpharmacological and pharmacological interventions to dampen its progression, where the SGLT2 inhibitors are clinically well-recognized drugs with cardio-renal benefits, as per DAPA-CKD and EMPA-CKD clinical trials^[1]^. But the molecular data supporting the anti-inflammatory mechanisms specifically in nondiabetic CKD patients remains unexplored. The current preclinical evidence supports their anti-inflammatory effects, which include downregulating cytokine release and modulating macrophage polarization from M1 (pro-inflammatory) to M2 (anti-inflammatory). Additionally, a reduction in CRP and other systemic markers is evident in diabetic CKD patients. According to a recent systematic review and meta-analysis on SGLT2 inhibitors, the post-hoc assessment of diabetes trials demonstrates the decline of hs-CRP and other systemic biomarkers of inflammation^[2]^. However, analogous data are limited to small studies in nondiabetic CKD cohorts. SGLT-2 inhibitors (dapagliflozin, empagliflozin) are the most prescribed oral antidiabetic drugs that have also demonstrated equivalent efficacy for reducing kidney events in patients with CKD irrespective of diabetes status. Nevertheless, large clinical trials are required for potential confirmation in nondiabetic CKD cohorts^[3]^.

Given the strong relation of chronic inflammation to CKD progression and cardiovascular events, we call for the prospective studies in well-phenotyped, nondiabetic CKD patients treated with SGLT2 inhibitors versus placebo, assessing the serial measurements of cytokines (TNFs, ILs) and systemic and urinary biomarkers (MCAP-1, NGAL), evaluating the phenotypic shift of macrophages and recognizing the gut–kidney axis to unveil whether inflammation suppression and immunomodulation underlie SGLT2 inhibitor-mediated effects in nondiabetic CKD cohorts and to elucidate this emerging therapeutic avenue^[1]^. This would help recognize patient subsets most likely to benefit and administer combination strategies with other anti-inflammatory therapies as well. The KDIGO 2024 guidelines emphasize understanding the non-traditional mechanisms of CKD, as current CKD management in various scenarios is still challenging, and **the inflammatory profile is yet to be fully explored.** Such studies will not only optimize patient selection but also open new therapeutic avenues for CKD management^[4]^. Thus, we urge the strong collaborations between nephrology and immunology researchers to design such trials for definitive testing of this inflammation hypothesis of SGLT2i-mediated renoprotection. Elucidating the complex interplay between SGLT2 inhibitors and inflammatory pathways in nondiabetic CKD patients holds profound implications for the development of targeted interventions, optimization of treatment regimens, and improvement of clinical outcomes, thereby potentially revolutionizing the management of this morbid condition.

TITAN Guidelines: This manuscript is in compliant to TITAN Guidelines, 2025, declaring no use of AI^[5]^.

## Data Availability

Not applicable.

## Peer and provenance statement

Not commissioned, externally peer-reviewed.

## References

[1] Zhang B Deng L. Application of SGLT-2 inhibitors in non-diabetic CKD: mechanisms, efficacy, and safety. Front Med (Lausanne) 2025;12. 10.3389/fmed.2025.1574693 PMC12259609 40665981

[2] Wang D Liu J Zhong L. The effect of sodium-glucose cotransporter 2 inhibitors on biomarkers of inflammation: a systematic review and meta-analysis of randomized controlled trials. Front Pharmacol 2022;13:1045235. 36467062 10.3389/fphar.2022.1045235 PMC9717685

[3] Trillini M Villa A Perna A. Randomized trial of dapagliflozin in patients with non-diabetic stage IV CKD. Kidney Int Rep 2025;10:3340–55. 41141510 10.1016/j.ekir.2025.07.020 PMC12545560

[4] Levin A Ahmed SB Carrero JJ. Executive summary of the KDIGO 2024 clinical practice guideline for the evaluation and management of chronic kidney disease: known knowns and known unknowns. Kidney Int 2024;105:684–701. 38519239 10.1016/j.kint.2023.10.016

[5] Agha R Mathew G Rashid R. Transparency in the reporting of Artificial INtelligence – the TITAN guideline. Prem J Sci 2025. doi:10.70389/PJS.100082

